# Kyste paratubaire tordu: à propos d'un cas rare de diagnostique difficile

**DOI:** 10.11604/pamj.2014.19.25.3417

**Published:** 2014-09-10

**Authors:** Saad Benkirane, Fatimazohra Fdili Alaoui, Hekmat Chaara, Hakima Bougern, Moulay Abdelilah Melhouf

**Affiliations:** 1 Service de Gynécologie Obstétrique, CHU Hassan II, Fès, Maroc

**Keywords:** Kyste paratubaire, torsion d′annexe, salpingectomie, paratubal cyst, adnexal torsion, salpingectomy

## Abstract

Les kystes para tubaires sont des lésions fréquentes, et peuvent être responsables de complications à type de torsion d'annexe qui est rarissime et difficile à diagnostiquer. Cette pathologie est souvent confondue à une torsion ovarienne, la prise en charge dans les deux cas nécessite une intervention chirurgicale en urgence afin de tenter de conserver l'annexe. Nous rapportons un cas rare d'une jeune patiente opérée d'un kyste para tubaire bénin tordue de diagnostic difficile.

## Introduction

Les kystes para tubaires sont des lésions fréquentes, dont la taille est le plus souvent modérée (< 6 cm) [1]. La majorité de ces formations sont bénignes mais plusieurs cas de dégénérescence sous forme d'adénocarcinomes [2] ont été décrits. Elles peuvent être responsables de complications rarissimes à type de torsion d'annexe qui est très difficile à diagnostiquer, d'hémorragie ou de rupture. Nous rapportons un cas rare d'une jeune patiente opérée d'un kyste para tubaire bénin tordue de diagnostique difficile

## Patient et observation

Mlle L.S âgée de 17 ans célibataire consulte pour des douleurs de la fosse iliaque droite aigue depuis 8H. Elle n'a aucun antécédent médical ou chirurgical notable, ses cycles sont réguliers et elle n'a jamais été sous traitement hormonal. Sa symptomatologie a débuté 18 H avant son admission par l'installation de douleur aigüe intense de la FID paroxystique et à type de torsion avec un seul épisode de vomissement, évoluant dans un contexte d'apyrexie et de conservation de l’état général. L'examen trouve une patiente stable sur le plan hémodynamique, apyrétique avec sensibilité au niveau de la fosse iliaque droite le toucher vaginal n'a pas été réalisé (patiente vierge), le toucher rectal trouve une masse latéro –utérinedroite trèssensibilité.

**Figure 1:**
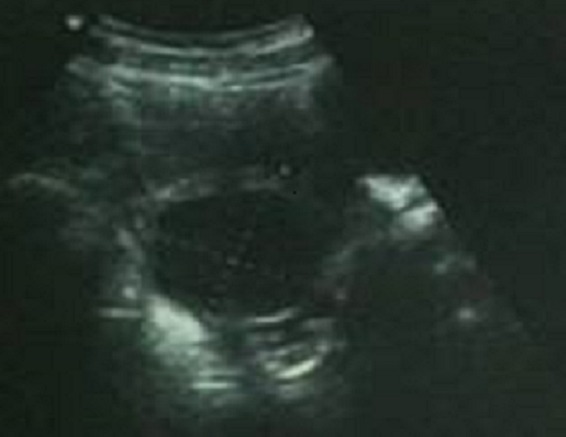
Image transonore a paroi fine sans végétation ni cloison mesurant 60/52 mm accoleé a l'ovaire droit et un épanchement de faible abundance

L’échographie pelvienne trouve un utérus de taille normale avec ligne d'interfacefine, ovaireGH visualisé avec présence d'une image transonore a paroi fine sans végétation ni cloison mesurant 60/52 mm accolée à l'ovaire droit, l'ovaire gauche est sans particularité, et un épanchement de faible abondance (Figure 1). La TDM montre un ovaire droit augmenté de taille mesurant 9cm siège d'un kyste simple de 6cm.

**Figure 2:**
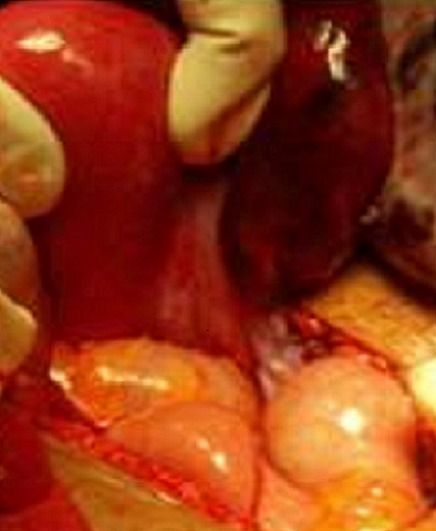
Image peropératoire objectivant un kyste paratubaire tordu avec trompe droite infarcie avec ovaire droit d'aspect normal

**Figure 3:**
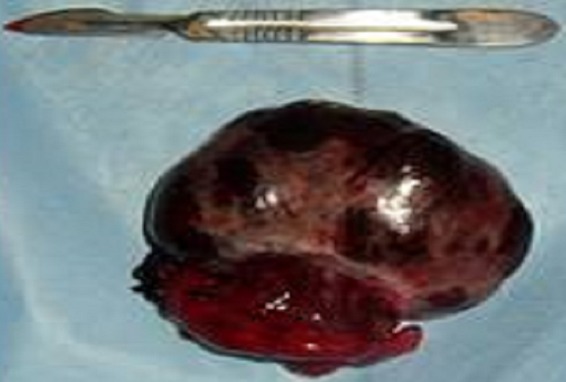
Pièce opératoire montrant une trompe et un kyste paratubaire infarcie

La patiente a bénéficié d'une exploration chirurgicale qui a objectiver un kyste para tubaire DT avec trompe DT tordus en 2 tours de spires sans atteinte de l'ovaire Dt, utérus,ovaire et trompe GH sont sans particularité avec présence d'un épanchement liquidien séreux de faible abondance. (Figure 2). Malgré la détorsion immédiate et le réchauffement au sérum salé,la trompe ne se revascularise pas d'où la décision d'une kystectomieDT avec salpingectomieDt rétrograde. (Figure 3). L'examen anatomopathologique définitif fait état d'un kyste para tubaire, sans signe suspect de malignité avec infarcissement de la trompe DT

## Discussion

Les kystes para tubaires sont des lésions très fréquentes, constituants environ 10% des masses annexielles [1]. Ils peuvent trouver leur origine embryologique dans des vestiges wolfienmésonéphrotiques, mullériensparamésonéphrotiques ou dans des inclusions mésothéliales [2]. L'hydatide de Morgagni est de loin la forme la plus fréquente des kystes d'origine paramésonéphrotique.ces kystes se présentent sous forme de kystes de taille variable au contact de l'ovaire [3]. Les kystes para tubaires sont souvent de découvertes fortuites et se compliquent très rarement: un faible pourcentage peut dégénérer en néoplasie (2% dans le travail deSTEIN et al). Il a été également rapporté de rares cas dans la littérature de torsion de kystes para tubaires entrainant avec eux une torsion de la trompe comme pour notre patente et dont le diagnostic précoce de torsion est primordial pour pouvoir sauver la trompe concernée sachant que les lésions ischémiques sont irréversibles après un délais de 36 à 48H [4],alors quand faut il y penser? La torsion d'un kyste para tubaire peut se manifester par des douleurs brutale de la fosse iliaque continues ou d'aggravation rapide, le toucher pelvien trouve une douleur latéralisée.

Le signe de WArneck (masse douloureuse et palpable lors de l'examen pelvien) correspondant au pédicule tordu est difficile à identifier surtout par le toucher rectale. Parfois des épisodes similaires mais spontanément résolutifs peuvent survenus dans les semaines précédentes.cette forme aigue est rare et amène rapidement à l'acte chirurgical, mais la torsion d'un kyste para tubaire se manifeste souvent sous forme subaigüe insidieuse avec une douleur progressive d'intensité initiale faible, s'aggrave lentement ce qui rend le diagnostic plus difficile comme ce qui est le cas pour notre patiente. Cette torsion se traduit en échographie par une formation anéchogène liquidienne pure en règle de taille moyenne (50 mm) au contact de l'ovaire. La palpation abdominale permet dans certain cas de dissocier le kyste de l'ovaire, ce qui permet d'en faire le diagnostic [5]. L’échographie doit systématiquement chercher à identifier les ovaires en dehors de la lésion Mais en pratique, il est bien difficile de distinguer un kyste paratubaire d′un kyste de l′ovaire lui-même à développement extra-ovarien, Le Doppler de la paroi du kyste ne permet pas, non plus, d′éviter ces faux-négatifs [6]. La trompe droite qui est libre dans le pelvis est préférentiellement atteinte, au contraire de la gauche qui est partiellement adhérente au sigmoïde

Enfin le traitement de la torsion tubaire isolée ne peut être que chirurgical. La coelioscopie est la technique de choix employée par la majorité des équipes. Le traitement va consister, si le diagnostic est précoce, en la détorsion de la trompe, le réchauffement au sérum physiologique et appréciation de la coloration [1]. En cas de non-recoloration, une salpingectomie sera pratiquée. L'attitude à l’égard de l'annexe controlatérale est très controversée, mais la fixation peut être préconisée, bien que la bi latéralisation soit rare [7]

## Conclusion

La torsion de kyste para tubaire est raricime et son diagnostic est très difficile, mais doit être systématiquement évoquer devant une adolescente consultant en urgence pour des douleurs pelviennes, surtout si des épisodes similaire se sont produits récemment.cette pathologie est souvent confondue avec une torsion ovarienne mais la prise en charge dans les deux cas nécessite une intervention chirurgicale en urgence et de préférence par coelioscopie afin de tenter de conserver l'annexe.
